# Trends in substance use admissions among older adults

**DOI:** 10.1186/s12913-017-2538-z

**Published:** 2017-08-22

**Authors:** Sumedha Chhatre, Ratna Cook, Eshita Mallik, Ravishankar Jayadevappa

**Affiliations:** 1 Department of Psychiatry, Perelman School of Medicine, University of Pennsylvania, 3535 Market Street, Suite 4051, Philadelphia, Pennsylvania 19104 USA; 2 Department of Medicine, Perelman School of Medicine, University of Pennsylvania, Philadelphia, Pennsylvania USA; 3 Department of Biotechnology, School of Engineering and Applied Sciences, University of Pennsylvania, Philadelphia, Pennsylvania USA; 4 Division of Urology, Department of Surgery, Perelman School of Medicine, University of Pennsylvania, Philadelphia, Pennsylvania 19104 USA; 5 Corporal Michael J. Crescenz VAMC, Philadelphia, Pennsylvania USA; 6 Leonard Davis Institute of Health Economics, University of Pennsylvania, Philadelphia, Pennsylvania USA; 7 Abramson Cancer Center, University of Pennsylvania, Philadelphia, Pennsylvania USA

**Keywords:** Substance abuse, Older adults, Treatment episode data set - admissions (TEDS-A), Trends in admission

## Abstract

**Background:**

Substance abuse is a growing, but mostly silent, epidemic among older adults. We sought to analyze the trends in admissions for substance abuse treatment among older adults (aged 55 and older).

**Methods:**

Treatment Episode Data Set - Admissions (TEDS-A) for period between 2000 and 2012 was used. The trends in admission for primary substances, demographic attributes, characteristics of substance abused and type of admission were analyzed.

**Results:**

While total number of substance abuse treatment admissions between 2000 and 2012 changed slightly, proportion attributable to older adults increased from 3.4% to 7.0%. Substantial changes in the demographic, substance use pattern, and treatment characteristics for the older adult admissions were noted. Majority of the admissions were for alcohol as the primary substance. However there was a decreasing trend in this proportion (77% to 64%). The proportion of admissions for following primary substances showed increase: cocaine/crack, marijuana/hashish, heroin, non-prescription methadone, and other opiates and synthetics. Also, admissions for older adults increased between 2000 and 2012 for African Americans (21% to 28%), females (20% to 24%), high school graduates (63% to 75%), homeless (15% to 19%), unemployed (77% to 84%), and those with psychiatric problems (17% to 32%).The proportion of admissions with prior history of substance abuse treatment increased from 39% to 46% and there was an increase in the admissions where more than one problem substance was reported. Ambulatory setting continued to be the most frequent treatment setting, and individual (including self-referral) was the most common referral source. The use of medication assisted therapy remained low over the years (7% - 9%).

**Conclusions:**

The changing demographic and substance use pattern of older adults implies that a wide array of psychological, social, and physiological needs will arise. Integrated, multidisciplinary and tailored policies for prevention and treatment are necessary to address the growing epidemic of substance abuse in older adults.

## Background

Substance abuse among older adults is one of the fastest growing health problems in the US [1–5]. The changing demographic composition of the older adult population in the US affects not only the prevalence of substance abuse, but also the need for a variety of services, including treatment. It is estimated that the number of older adults who will need treatment for substance abuse will increase from 1.7 million in 2000–2001 to 4.4 million in 2020 [3, 6]. This increase is partially attributed to the aging baby boomer population who has had more exposure to drugs, alcohol and tobacco from a younger age, which is reported to be a risk factor for use and abuse of these substances in later years [6–8] . The use of illicit drugs among older adults appears to be increasing. A study showed that the use of illicit drugs among adults age 50–59 almost doubled between 2002 and 2007 (5.1% to 9.4%). Also, of the adults aged 50–59 who were using illicit drugs in 2007, almost 90% had started using them before age 30. This implies lifelong nature of illicit drug use [9]. Analysis of the 2008 data from the Drug Abuse Warning Network surveillance system showed that of the 1.1 million emergency department episodes for adverse drug reactions, 61% were for persons aged 65 or older. Also, almost 25% these episodes were due to adverse reactions to central nervous system drugs [10]. Although limited, research indicates important racial and ethnic differences in the prevalence of substance abuse in older adults. Among persons aged 65–74, being white, male, and divorced or widowed was associated with higher odds of lifetime alcohol use disorder [11]. Being African American or Hispanic was one of the several factors associated with sub-threshold alcohol dependence in the past year [12]. In one study, African Americans aged 55 and older were reported to have higher prevalence as well as higher rates of treatment admissions for illicit drugs such as cocaine [13]. Important gender differences in the older persons with substance abuse are also noted. Women make-up a larger portion of the older population, especially among those aged 85 and older. The pattern of substance use in older women is different than that of older men. Research indicates that binge drinking in women aged 65 and older is lower compared to their male counterparts [14]. Also, older women had lower rates for alcohol dependence or abuse, drug dependence or abuse or both conditions, and lower past-year use of illicit drugs, compared to older men (1.4% vs. 2.2% in 2010) [10].

Even as the number of older adults with substance abuse is on the rise, substance abuse is often undetected and undertreated in this population [15, 16]. Due to the stigma attached to substance abuse, elderly patients may not report this issue [17, 18]. Therefore, the true prevalence of substance abuse in this population remains unknown. In addition, providers are often too busy or may confuse the symptoms of substance abuse disorders with other co-morbidities, age-related changes or reactions to stressful life situations [17, 19]. Number of co-morbidities increase with age and presence of substance abuse can lead to worsening of medical consequences and outcomes of care [19]. Of the total spending for substance abuse disorder treatments, a substantial share is borne by public sources: Medicare, Medicaid, local, state and federal governments. For example, in 2009, 69% of spending on substance abuse treatment came from public sources [20]. One study reported that compared to younger adults, the proportion of older adults seeking treatment for illicit drugs abuse for the first time is on the rise [2].

Thus, a rise in substance abuse among older persons coupled with the aging of the US population has strong implications for treatment demands on the healthcare system. Though the definition of an ‘older adult’ may vary slightly, there is consensus that interaction of age related changes (physiological, psychological, functional or social) and substance abuse is detrimental to the wellbeing and exerts significant burden on the healthcare system. Following the criteria used by several prior studies, in this study we define ‘older adults’ as those aged 55 or older [2, 21–23]. Information about trends in substance abuse pattern among older adults is essential for developing appropriate preventive and treatment policies and for resource planning. Objective of this study was to analyze the national trends in admissions of older adults (aged ≥55 years) to publically funded substance abuse treatment facilities between 2000 and 2012. We analyzed the demographic attributes, characteristics of the substance abused, age at first use and type of admission for the cohorts of older adult admissions between 2000 and 2012.

## Methods

### Data source

We used the Treatment Episode Data Set - Admissions (TEDS-A)**,** an administrative public use data system that is maintained and sponsored by the Center for Behavioral Health Statistics and Quality at the Substance Abuse and Mental Health Services Administration (SAMHSA) [24]. All public and private substance abuse treatment facilities that receive public funds are required to report the information about annual flow of admissions via state funding agency to TEDS-A. In TEDS-A, the unit is analysis is an admission. This database also includes information on demographic characteristics (age, race, gender, employment, education, pregnancy, veteran status, health insurance), substance abuse behavior (type of substance, mode of use, frequency of usage, age at first use), treatment characteristics (referral source, prior treatment, service setting), geographic information (region, division), and presence of psychiatric diagnosis at each admission.

### Data analysis

Our analysis included all admissions to the publically funded substance abuse treatment programs between 2000 and 2012 for persons who were aged 55 years or older at the time of admission**.** The unit of analysis is an admission and a person can have multiple admissions in a year. However, it is not possible to identify an individual person and thus dependence of observations cannot be adjusted for. Given the large sample size, a stringent criteria of *p* < .0001 was adopted for determining statistical significance of Chisq tests. Substance use treatment admissions among older adults as a proportion of total admissions were compared over time. We also compared the trends in admission for following primary substances: alcohol, cocaine/crack, heroin, marijuana/hashish, nonprescription methadone, other opiates and synthetics, methamphetamine, benzodiazepines, Phencyclidine, other hallucinogens, other amphetamines, other stimulants, other non-benzodiazepine, other non-barbiturates sedatives or hypnotics, inhalants, over-the-counter medications and other.

In addition to demographic characteristics, substance characteristics, including the number of substances abused at the time of admission, service treatment setting, referral source, number of prior treatment admission episodes, and use medication assisted therapy were analyzed for cohorts of older adult admissions. Finally, we analyzed the trend in type of substance abuse, and age at first initiation for those with no prior treatment admissions vs. those with at least one prior treatment admission.

### Study results

Of the total admissions to publically funded substance abuse treatment programs in year 2000, 3.4% (*n* = 60,112) were for older adults. There was a steady increase of this proportion over time, and in 2012, admissions for older adults accounted for 7.0% (*n* = 121,015) of all admissions. At the same time, the total number of admissions for substance abuse combined for all age-groups changed only slightly. Demographic characteristics of cohorts of older adult admissions between 2000 and 2012 are presented in Table 1. Overall, most admissions from 2000 to 2012 were among non-Hispanic white, male, unmarried, high school graduates, unemployed and those with housing. However, some of these variables showed changes over time. For example, admissions for older adults increased between 2000 and 2012 for African Americans (21% to 28%), females (20% to 24%), high school graduates (63% to 75%), unmarried (79% to 84%), homeless (15% to 19%), unemployed (77% to 84%), and those with psychiatric problems (17% to 32%).

**Table 1:** Demographics of substance use treatment admissions in older adults, TEDS-A 2000–2012

Characteristic	2000 (*N* = 60,112) n (%)	2004 (*N* = 69,310) n (%)	2008 (*N* = 104,431) n (%)	2012 (*N* = 121,015) n (%)
Gender*				
Male	48,155(80.4)	54,320(78.4)	80,344(76.95)	91,255(75.54)
Female	11,742(19.6)	14,963(21.6)	24,071(23.05)	29,546(24.46)
Race/ethnicity*				
White non-Hispanic	35,071(58.34)	39,219(56.58)	57,669(55.22)	67,068(55.42)
Black Non-Hispanic	12,874(21.42)	16,467(23.76)	27,630(26.46)	34,258(28.31)
Hispanic	9835(16.36)	10,973(15.83)	14,743(14.12)	14,371(11.88)
Other	2332(3.88)	2651(3.2)	4389(4.20)	5318(4.39)
Marital status*				
Married	12,384(20.6)	14,017(20.22)	18,673(17.88)	19,748(16.32)
Not married	47,728(79.4)	55,293(79.78)	85,758(82.12)	101,267(83.68)
Education*^a^				
Completed high school	36,545(62.99)	46,759(70.22)	75,480(73.98)	88,641(74.8)
Did not complete high school	21,470(37.01)	19,829(29.78)	26,546(26.02)	29,857(25.2)
Employment*^b^				
Employed	12,959(22.97)	13,999(21.37)	20,756(20.15)	18,326(15.55)
Not employed	43,465(77.03)	51,499(78.63)	82,261(79.85)	99,544(84.45)
Living arrangement*^c^				
Homeless	6844(14.55)	9689(16.93)	17,441(17.48)	22,344(18.69)
Not homeless	40,201(85.45)	47,536(83.07)	82,334(82.52)	97,238(81.31)
Other psychiatric illness*^d^				
No	31,000(83.06)	31,486(75.59)	49,887(70.57)	57,743(67.62)
Yes	6321(16.94)	10,169(24.41)	20,805(29.43)	27,655(32.38)

*p-value <0.0001; a 2.7% missing; b 3% missing; c 9.75% missing; d 34% missing

**Fig. 1:**
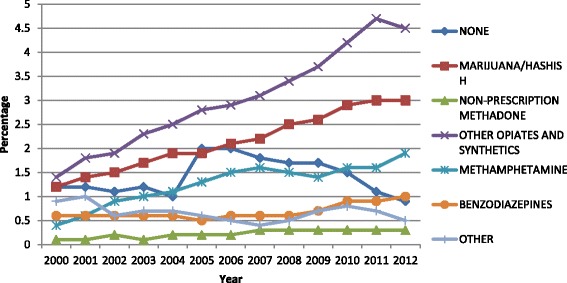
Percent of admissions with specific substance as the primary substance of abuse of those aged ≥ 55

In Table 2, we present the type of substance that caused the treatment admission and other characteristics of the admission. Among our cohorts of older adult admissions, majority of the admissions were for alcohol as the primary substance. However, there was a decreasing trend in this proportion from 2000 to 2012 (77% to 64%). On the other hand, proportion of admissions for following primary substances showed steep increase between 2000 and 2012: cocaine/crack (63% increase), marijuana/hashish (150% increase), heroin (26% increase), non-prescription methadone (200% increase), other opiates and synthetics (221% increase), and benzodiazepines (67% increase) (Table 2 and Fig. 1). For the base year (i.e., year 2000), the proportion of admissions for the following substance was under 1% and therefore not reported in Table 2: nonprescription methadone, methamphetamine, benzodiazepines, Phencyclidine, other hallucinogens, other amphetamines, other stimulants, other non-benzodiazepine, other non-barbiturates sedatives or hypnotics, inhalants, over-the-counter medications and other. The proportion of admissions for poly-substances grew over time (20% in 2000 vs. 38% in 2012). Poly-substance is defined as use of secondary and/or tertiary problem substances, in addition to the primary substance as reported at the time of admission.

**Table 2:** Substance use and treatment characteristics across substance use treatment admission episodes in older adults, TEDS-A 2000–2012

Characteristic	2000 *N* = 60,112 n (%)	2004 *N* = 69,310 n (%)	2008 *N* = 104,431 n (%)	2012 *N* = 121,015 n (%)
Number of substances*				
1	46,227(76.9)	48,308(69.7)	67,689(64.82)	72,934(60.27)
2	8843(14.71)	13,563(19.57)	24,655(23.61)	32,791(27.1)
3	3349(5.57)	5516(7.96)	9933(9.51)	13,839(11.44)
Primary substance problem*				
Alcohol	45,527(77.05)	47,185(69.8)	67,247(64.61)	78,003(64.68)
Heroin	6912(11.7)	9395(13.9)	15,802(15.18)	17,896(14.84)
Cocaine/crack	2865(4.85)	5112(7.56)	9394(9.03)	9449(7.84)
Other opiates and synthetics	799(1.35)	1696(2.51)	3580(3.44)	5470(4.54)
Marijuana/hashish	721(1.22)	1270(1.88)	2561(2.46)	3624(3.01)
Any substance use type				
Alcohol only	39,675 (66.0) 9018	38,477 (55.51)	51,585 (49.40)	54,961 (45.42)
Other drug only	(15.0)	14,369 (20.73)	25,198 (24.13)	29,680 (24.53)
Alcohol and other drug	9726 (16.18)	14,541 (20.98)	25,494 (24.41)	34,923 (28.86)
Service setting				
Detox	19,463(32.38)	20,048 (30.17)	27,759 (26.59)	11,096 (30.92)
Rehab	7659(12.74)	9398 (13.57)	15,803 (15.14	18,162 (15.01)
Ambulatory	32,981 (54.87)	39,847 (57.51)	60,849 (58.28)	65,425 (54.05)
Referral source*				
Individual (includes self-referral)	23,505(40.62)	27,433(40.73)	41,307(40.37)	51,308(43.27)
Healthcare provider (Alcohol/drug abuse /other)	14,366 (24.83)	15,225 (22.6)	22,642 (22.13)	25,021(21.1)
School (educational)/Employer/EAP	791(1.37)	892(1.33)	995(0.97)	848(0.71)
Other community referral	3761(6.5)	5862(8.7)	9887(9.66)	12,823(10.81)
Criminal justice	15,438(25.68)	17,943(25.89)	27,495(26.33)	28,584(23.62)
Number of prior episodes*^a^				
0	20,503(42.98)	22,573(41.45)	36,092(39.85)	36,584(35.18)
≥ 1	27,198 (57.02)	31,888 (58.55)	54,489(60.15)	67,410(64.82)
Medication assisted therapy used*^b^ Yes	4541(7.85)	5394(8.41)	9520(9.76)	10,792(9.35)

*p-value <0.0001; a 17.6% missing; b 5.6% missing

Table 2 also presents the treatment characteristics of the cohorts of older adult admissions between 2000 and 2012. The majority of the admissions were to ambulatory setting (54%) and individual (including self-referral) was the most common referral source (40% - 43%). The use of medication assisted therapy remained low over the years (7% - 9%). The number of Admissions with prior history of substance abuse treatment increased from 39% to 46%. In year 2000, admissions where alcohol was the only substance reported accounted for two-third of all older adult admission. However, by 2012, less than half of all admissions were for alcohol only. At the same time, the proportion of admissions for other drugs only and alcohol plus other drugs increased between 2000 and 2012.

In Table 3, we present the comparison of substance abuse type across two sub-groups: those without prior admissions vs. those with at least one prior admission. Among those older adults without prior admissions, the proportion of admissions for alcohol only declined (70% in 2000 vs. 52% in 2012). On the other hand, the proportion of admissions for other drugs only and those for alcohol plus other drugs almost doubled between 2000 and 2012. A similar pattern was observed for those with at least one prior admission. Age at first initiation also showed comparable trends between these two sub-groups (data not shown). When the admission was for alcohol only or for alcohol with drug, more than 80% reported age at first use as younger than 25%. However, when the admission was for other drugs only, only half reported first use at age younger than 25 years thus indicating continued initiation of drug use over the adult years, including 55 or older.

**Table 3:** Substance abuse type for older adults by number of prior admissions

Substance abuse type**	No prior admission ^*^				One or more prior admissions^*^			
	2000 (*n* = 20,503)	2004 (*n* = 22,573)	2008 (*n* = 36,092)	2012 (*n* = 36,584)	2000 (*n* = 39,609)	2004 (*n* = 46,737)	2008 (*n* = 68,339)	2012 (*n* = 84,431)
Alcohol only	14,376	13,497	19,868	19,155	25,299	24,530	31,717	35,906
	(70.1)	(61.8)	(*n* = 55.1)	(*n* = 52.5)	(63.9)	(52.5)	(46.4)	(42.4)
Other drugs only	2478	4005	7919	8719	6540	10,364	17,279	20,961
	(12.1)	(17.7)	(21.9)	(23.8)	(16.5)	(22.2)	(25.3)	(24.8)
Alcohol and other drugs	2615	3702	7136	8260	7111	10,839	18,358	26,663
	(12.7)	(16.4)	(19.8)	(22.6)	(17.9)	(23.2)	(26.9)	(31.6)
None	1034	919	1169	450	659	1004	985	1001
	(5.0)	(4.1)	(3.2)	(1.2)	(1.7)	(2.1)	(1.4)	(1.2)

**Primary, secondary or tertiary substance of abuse reported at the time of admission

*p-value <0.0001

## Discussion

Substance abuse is an important psychosocial comorbidity in older adults and our results add to the growing body of evidence that the magnitude of substance abuse disorders among older adults is escalating [1–3, 5, 6, 22, 25, 26]. Longitudinal data from TEDS-A demonstrate that while the total number of admissions to the publically funded substance abuse treatment programs have stayed almost constant between 2000 and 2012, there is an increase in the proportion of admissions attributable to older adults. Our results also show that initiation of drug use is spread over the lifespan of the older adult as opposed to first use of alcohol which happened mostly prior to age 25. Socio-cultural factors appear to have a role in the observed variations in prevalence, type of substance abused, treatment characteristics and outcomes for substance abuse. Thus, the issue of substance abuse among older adults may be viewed in a broad sociocultural framework.

Several demographic and service related factors may have contributed to the changes in demographic composition and treatment characteristics of the substance abusing older adults. One important demographic factor is the aging baby boomer cohort (those born between 1946 and 1964). The baby boomer cohort turns 55 years old between 2001 and 2019, and 65 years old between 2011 and 2029. In addition to being larger in numbers, this cohort also has higher prevalence of lifetime substance use, compared to earlier older cohorts [6, 27]. In addition, a history of alcohol abuse increases the risk of substance use in late life [26]. Thus, some of the observed growth in number of older adult admissions to substance abuse treatment program may be a reflection of aging of the baby boomer cohort. Although alcohol remains the top primary reason for admission among older adults, the number of admissions where alcohol was either the primary or the only substance abused have decreased between 2000 and 2012. On the other hand, there was a significant increase in the proportion of admissions for drug use only or for combined drug and alcohol use.

Source of referral also offers some interesting insight into the changing composition of older adult admissions to substance abuse treatment programs. Referrals from other community sources increased between 2000 and 2012. However, overall referrals by healthcare providers declined over the study period, 24% in 2000 vs. 21% in 2012. Also, the overall referrals made by criminal justice system declined over time. This result is similar to the one reported by a study of TEDS-A for the period between 1992 to 2005 [22]. While increase in community referrals and individual referrals suggests better awareness and access to substance abuse treatments, the decrease in referrals from providers is indicative of lost-opportunity for screening and referrals [21]. Time pressure, lack of training and mistaking substance abuse symptoms for those associated with normal aging may be some of the reasons for the decrease in referrals from providers.

Medication assisted therapy is commonly used in treatment for alcohol and opioid-related addictions. However, despite research demonstrating the effectiveness of medication assisted therapy as an evidence-based practice for substance abuse, such treatment remains underutilized. We observed only 7.9–9.8% of total admissions in older adults reporting medication assisted opioid therapy as part of the treatment plan. A separate analysis (data not reported) showed that for admissions where other drugs (and not alcohol) was the substances abused, 43% of admission had medication assisted opioid therapy as part of the treatment plan, however by year 2012, this proportion decreased to 31%. Additionally, medication assisted therapy was reported by a very small proportion of admissions that were for alcohol plus other drug abuse. Another study has reported that less than one-half of the 2.5 million Americans aged 12 or older who abused or were dependent on opioids in 2013 received medication assisted therapy [28]. In our study, admissions for older adults where primary substance was opiates and other synthetics increased by 221% between 2000 and 2012. It is not surprising that opiates, which are the most commonly prescribed drug class in the US, have shown the most increase in admissions related to substance abuse. The nearly 9-fold increase in opioid prescriptions from office based medical visits by older adults that occurred between 1995 and 2010 suggests that physicians have pursued greater pain control in this group [20]. Additionally it has been shown that overdose deaths involving opioid analgesics now exceed deaths involving heroin and cocaine combined [5].

We note certain limitations to our study that are intrinsic to the TEDS-A data. First and foremost, the unit of analysis in TEDS-A data is an admission, and not a person. It is difficult to determine if the increase in number of admissions is due to an increase in the number of unique older adults seeking treatment, or is a reflection of multiple admissions made by a smaller group of older adults. To some extent, we addressed this limitation by analyzing the number of prior visits in order to isolate those new to the system (no prior admissions) versus those who have repeat admissions (at least one prior admission). Secondly, TEDS does not include non-publically funded substance abuse treatment programs or data from the Department of Veterans Administration. The demographic and substance abuse profile of those not seeking care in a publically funded substance abuse program may be different than that observed in TEDS-A, and this may affect the generalizability of the results. As the admissions reported in TEDS-A are from publically funded substance use programs, they are affected by peripheral factors such as the availability of funds, target groups, and state policies. Additionally, the self-reported information in TEDS has potential for memory and/or personal biases. Finally, completeness of data reporting may differ by state and may lead to variation in the magnitude of available data. Thus, interpretations of our results must be made within the context of these data limitations.

### Policy implications

Our findings have several policy implications. First, our results provide an indication of service needs that is essential for planning purposes. The healthcare system must be prepared to treat a large number of older adults with substance abuse resulting from a growth in the number of older adults coupled with the growing substance abuse epidemic in this population. The changing composition of this cohort implies a wide array of psychological, social, and physiological needs that must be addressed in the coming years. For example, as older adults often come in contact with their primary care or other healthcare providers, it is important that these providers are able to distinguish substance abuse problems from physical or mental health problems, and refer the patients to treatment as needed. Given the challenges in diagnosing substance abuse in aging elderly, policies that support geriatric education and training for healthcare providers and others working with the aging population (for e.g. aging service providers) may be beneficial. Additionally, the type of substance abused and treatment setting presents specific challenges to publically funded treatment programs, including Medicare and Medicaid. The Affordable Care Act includes many provisions to improve and expand treatment for people with substance abuse disorders, and also expands the Medicaid programs in certain states. Therefore, the issue of substance abuse among older adults can be effectively addressed by integrated and multidisciplinary collaboration among the treatment community and other service systems for aging adults.

## Conclusions

Substance abuse among older adults is a serious issue and its magnitude will grow with the aging of the baby boomer cohort. Even though the total number of substance abuse admissions between 2000 and 2012 remained mostly unchanged, the proportion of admissions attributable to older adults increased more than two-fold. While alcohol still remains the most frequent reason for the admission to substance abuse treatment, this proportion is declining. On the other hand, cocaine and heroin related admissions (i.e., where cocaine or heroin was the client’s primary substance problem) are on the rise in the older adult population.

Substance abuse translates into treatment need, may affect health outcomes and complicate the treatment of other comorbid conditions among older persons. Majority of the substance abuse policies focus on younger population. For example, heroin is usually considered as a problem of the younger population and research indicates that mortality is high in heroin users [2]. However, considerable heroin usage among older adults was observed and it has grown over time*.* Thus, there is an immediate need for specific and tailored strategies for screening, linkage to care and retention in care for older substance abusers. Research shows that once in treatment, older adults respond well to care [29]. Older adults with substance abuse experience higher prevalence of co-occurring mental health, and general health related comorbidities. Also their drug use initiation and experience appears to span over the entire age spectrum. A coordinated and integrated approach that brings together health professionals and community resources can facilitate early identification of substance use, and linkage to and retention in care.

## Abbreviation

TEDS: Treatment episode data set
